# Supplementary material for: The Social Vulnerability Index, Mortality and Disability in Mexican Middle-Aged and Older Adults
Source: Geriatrics (Basel). 2021 Mar 8;6(1):24. doi: 10.3390/geriatrics6010024 (PMC8006046; doi:10.3390/geriatrics6010024)
Supplement: Supplementary file 1 [file geriatrics-06-00024-s001.pdf]

Supplementary table 1: The social vulnerability index

Variable	Description/Question	Codification	N (%)	Mean (±SD)
Communication	Knows how to write and read a message	1=No	12,276 (86.7)	0.13 (0.3)
		0=Yes	1,883 (13.3)	
Couple	Is married or has a couple	1=No	4,260 (29.7)	0.2 (0.4)
		0=Yes	10,069 (70.3)	
Loneliness	Feels lonely even if he/she is not alone	1=Yes	4,322 (30.1)	0.3 (0.5)
		0=No	10,007 (69.8)	
Couple understanding	How much does your couple understand your feelings about things?	1=No couple	4,260 (29.8)	0.37 (0.43)
		0.6=Nothing	545 (3.8)	
		0.3=Something	2,422 (16.9)	
		0=A lot	7,069 (49.4)	
Couple reliability	How much can you confide in your couple if you have a serious problem?	1=No couple	4,260 (29.8)	0.35 (0.44)
		0.3=Something	466 (3.2)	
		0.6=Nothing	1,460 (10.2)	
		0=A lot	8,122 (56.7)	
Couple listening	How much does your couple listen if you need to talk about your worries?	1=No couple	4,260 (29.8)	0.37 (0.43)
		0.3=Something	607 (4.2)	
		0.6=Nothing	1,973 (13.7)	
		0=A lot	7,464 (52.2)	
Couple disappointment	How often does your couple disappoint you when you are counting on her/him?	1=No couple	4,260 (29.8)	0.45 (0.41)
		0.6=A lot	2,200 (15.4)	
		0.3=Something	2,431 (17.1)	
		0=Nothing	5,351 (37.6)	

Children understanding	How much do your children understand your feelings about things?	1=No children	800 (5.6)	0.15 (0.2)
		0.3=Something	555 (3.9)	
		0.6=Nothing	2,997 (21)	
		0=A lot	9,892 (69.4)	
Children reliability	How much can you confide in your children if you have a serious problem?	1=No children	800 (5.6)	0.12 (0.26)
		0.3=Something	492 (3.4)	
		0.6=Nothing	2,143 (15)	
		0=A lot	10,826 (75.9)	
Children listening	How much do your children listen if you need to talk about your worries?	1=No children	800 (5.6)	0.14 (0.27)
		0.6=Nothing	612 (4.3)	
		0.3=Something	2,558 (17.9)	
		0=A lot	10,286 (72.1)	
Children disappointment	How often do your children disappoint you when you are counting on her/him?	1=No children	800 (5.6)	0.26 (0.31)
		0.6=A lot	2,858 (20.1)	
		0.3=Something	3,283 (23.1)	
		0=Nothing	7,248 (51.1)	
Friends understanding	How much do your friends understand your feelings about things?	1=No friends	5,497 (33.5)	0.49 (0.43)
		0.6=Nothing	788 (5.5)	
		0.3=Something	3,154 (22.2)	
		0=A lot	4,765 (33.5)	
Friends reliability	How much can you confide in your friends if you have a serious problem?	1=No friends	5,497 (33.5)	0.51 (0.42)
		0.6=Nothing	1,067 (7.5)	
		0.3=Something	3,286 (23.1)	
		0=A lot	4,391 (30.8)	

Friends listening	How much do your friends listen if you need to talk about your worries?	1=No friends	5,497 (33.5)	0.5 (0.43)
		0.3=Something	955 (6.7)	
		0.6=Nothing	3,073 (21.6)	
		0=A lot	4,723 (33.1)	
Friends disappointment	How often do your friends disappoint you when you are counting on her/him?	1=No friends	5,497 (38.7)	0.51 (0.42)
		0.6=A lot	1,262 (8.9)	
		0.3=Something	3,007 (21.2)	
		0=Nothing	4,429 (31.2)	
Planning future	There's no sense in planning a lot for the future	1=Agree	5,602 (39.7)	0.48 (0.46)
		0.6=Somewhat agree	1,526 (10.8)	
		0.3=Somewhat disagree	827 (5.8)	
		0=Disagree	6,133 (43.5)	
Good things	The really good things that happen to one are mostly due to luck	1=Agree	5,426 (38.4)	0.5 (0.44)
		0.6=Somewhat agree	2,107 (14.9)	
		0.3=Somewhat disagree	1,109 (7.8)	
		0=Disagree	5,470 (38.7)	
Responsible in success	One is responsible for one's own successes	1=Disagree	207 (1.4)	0.04 (0.15)
		0.6=Somewhat disagree	185 (1.3)	
		0.3=Somewhat agree	793 (5.5)	
		0=Agree	13,017 (91.6)	
Do anything	One can do just about anything she/he put her/his mind to	1=Disagree	333 (2.3)	0.06 (0.1)
		0.6=Somewhat disagree	275 (1.9)	
		0.3=Somewhat agree	1,062 (7.4)	
		0=Agree	12,538 (88.2)	

Bad luck	Most of one's problems are due to bad luck	1=Agree	3,144 (22.2)	0.32 (0.41)
		0.6=Somewhat agree	1,465 (10.3)	
		0.3=Somewhat disagree	1,380 (9.7)	
		0=Disagree	8,121 (57.5)	
Bad things	One has little control on the bad things that happen to her/him	1=Agree	6,658 (47.5)	0.62 (0.41)
		0.6=Somewhat agree	2,447 (17.4)	
		0.3=Somewhat disagree	1,321 (9.4)	
		0=Disagree	3,588 (25.6)	
Misfortunes	One's misfortunes are the result of one's own mistakes	1=Disagree	2,155 (15.2)	0.24 (0.36)
		0.6=Somewhat disagree	854 (6.1)	
		0.3=Somewhat agree	2,233 (15.8)	
		0=Agree	8,864 (62.8)	
Failures	One is responsible for one's own failures	1=Disagree	298 (2.1)	0.04 (0.17)
		0.6=Somewhat disagree	168 (1.1)	
		0.3=Somewhat agree	829 (5.8)	
		0=Agree	12,904 (90.8)	
Ideal	In most things, my life is close to my ideal	1=Disagree	1,714 (12.2)	0.2 (0.34)
		0.5=Neutral	2,263 (16.1)	
		0=Agree	10,065 (71.6)	
Excellent conditions	The conditions of my life are excellent	1=Disagree	1,851 (13)	0.26 (0.35)
		0.5=Neutral	3,724 (26.2)	
		0=Agree	8,631 (60.7)	
Life satisfaction	I am satisfied with my life	1=Disagree	776 (5.4)	0.1 (0.26)
		0.5=Neutral	1,405 (9.8)	

		0=Agree	12,070 (84.7)	
Important things	So far, I have gotten the things that are important to me in my life	1=Disagree	949 (6.6)	0.13 (0.28)
		0.5=Neutral	1,801 (12.6)	
		0=Agree	11,471 (80.6)	
Change nothing if born again	If I were to be born again, I would change almost nothing of my life	1=Disagree	2,687 (19)	0.26 (0.39)
		0.5=Neutral	2,232 (15.8)	
		0=Agree	9,203 (65.1)	
Technology for communication	During the past year did you talk on the phone with relatives or friends or use the computer to send messages or use the internet?	1=No	4,460 (31.1)	0.31 (0.46)
		0=Yes	9,861 (68.8)	
Caregiving	During the past year did you care for a sick or disabled adult?	1=No	11,739 (81.9)	0.81 (0.38)
		0=Yes	2,581 (18)	
Babysit	During the past year did you care for children under 12 years old?	1=No	10,462 (73)	0.73 (0.4)
		0=Yes	3,862 (26.9)	
Volunteer	During the past year did you volunteer work or support an organization without pay or reward?	1=No	13,016 (90.9)	0.9 (0.28)
		0=Yes	1,299 (9.1)	
Classes	During the past year did you attend a training course, lecture or class?	1=No	11,974 (83.6)	0.83 (0.37)
		0=Yes	2,344 (16.3)	
Club	During the past year did you attend a sporting or social club?	1=No	13,296 (92.8)	0.92 (0.25)
		0=Yes	1,026 (7.1)	
Read	During the past year did you read a book, magazine or newspaper?	1=No	5,797 (40.5)	0.4 (0.49)
		0=Yes	8,494 (59.4)	
Crosswords	During the past year did you do crosswords, puzzles, jigsaw puzzles or Sudoku?	1=No	11,840 (82.7)	0.82 (0.37)
		0=Yes	2,467 (17.2)	
Card games		1=No	11,998 (83.8)	0.83 (0.36)

	During the past year did you play games such as cards, dominoes, or chess?	0=Yes	2,308 (16.1)	
Maintenance	During the past year did you do activities having to do with home maintenance, repairs, gardening, etc.?	1=No	6,496 (45.3)	0.45 (0.49)
		0=Yes	7,824 (54.6)	
Television	During the past year did you watch television?	1=No	1,285 (8.9)	0.08 (0.28)
		0=Yes	13,039 (91)	
Crafts	During the past year did you sew, embroider, knit or other crafts?	1=No	10,170 (71)	0.71 (0.45)
		0=Yes	4,153 (29)	
Financial status	Would you say your financial situation is…?	1=Poor	1,859 (12.9)	0.71 (0.16)
		0.75=Fair	9,248 (64.6)	
		0.5=Good	2,921 (20.4)	
		0.25=Very good	192 (1.3)	
		0=Excellent	96 (0.6)	
Scholar	Number of years in school	Inverse reciprocal of years in school		0.73 (0.21)

Supplementary table 2: The frailty index

Variable	Description/Question	Codification	N (%)	Mean (±SD)
Self-rated health	Would you say your health is…	1=Poor	1,812 (12.6)	0.6 (0.21)
		0.75=Fair	7,271 (50.7)	
		0.5=Good	4,249 (29.6)	
		0.25=Very good	637 (4.4)	
		0=Excellent	357 (2.4)	
Compared to 2 years ago self-rated health	Comparing your health now with your health two years ago, would you say your health now is…	1=Much worse	534 (3.7)	0.53 (0.19)
		0.75=Somewhat worse	3,707 (25.8)	
		0.5=More or less the same	8,055 (56.2)	
		0.25=Somewhat better	1,497 (10.4)	
		0=Much better	535 (3.7)	
Compared to others health	Compared with other people your age, would you say that currently your health is…	1=Worse	1,591 (11.2)	0.38 (0.31)
		0.5=Better	7,734 (54.5)	
		0=More or less the same	4,857 (34.2)	
Hypertension	Has doctor or medical personnel ever diagnosed you with hypertension or high blood pressure?	1=Yes	6,164 (43.1)	0.43 (0.49)
		0=No	8,133 (56.8)	
Cancer	Has doctor or medical personnel ever diagnosed you with cancer?	1=Yes	303 (2.1)	0.02 (0.14)
		0=No	14,003 (97.8)	
Respiratory diseases	Has doctor or medical personnel ever told/diagnosed you with a respiratory illness, such as asthma or emphysema?	1=Yes	838 (5.8)	0.05 (0.23)
		0=No	13,465 (94.1)	
Stroke	Has doctor or medical personnel told you that you had a stroke?	1=Yes	283 (1.9)	0.01 (0.13)
		0=No	14,031 (98)	
Arthritis		1=Yes	1,942 (13.5)	0.13 (0.34)

	Has doctor or medical personnel ever diagnosed you with arthritis or rheumatism?	0=No	12,357 (86.4)	
Tuberculosis	In the last 2 years, has doctor or medical personnel told you that you have tuberculosis?	1=Yes	35 (0.2)	0.002 (0.04)
		0=No	14,262 (99.8)	
Pneumonia	In the last 2 years, has doctor or medical personnel told you that you have pneumonia?	1=Yes	219 (1.6)	0.01 (0.12)
		0=No	14,086 (98.4)	
Herpes zoster	In the last 2 years, has doctor or medical personnel told you that you have herpes zoster?	1=Yes	266 (1.9)	0.01 (0.13)
		0=No	14,028 (98.1)	
Falls	Have you fallen down (how many times) in the last two years?	1=More than 1	3,569 (24.9)	0.31 (0.42)
		0.5=One	2,028 (14.1)	
		0=None	8,726 (60.9)	
Adulthood fracture	In the last ten years, have you fractured any bone(s) including your hip?	1=Yes	1,445 (10.3)	0.1 (0.3)
		0=No	12,516 (89.6)	
Vision	How is your vision (with glasses)?	1=Poor/legally blind	1,043 (7.4)	0.58 (0.2)
		0.75=Fair	5,045 (36.1)	
		0.5=Good	6,151 (43.9)	
		0.25=Very good	1,156 (8.2)	
		0=Excellent	594 (4.2)	
Hearing aid	Do you usually use a hearing aid or auditory device?	1=Yes	165 (1.1)	0.01 (0.1)
		0=No	14,162 (98.8)	
Pain	Do you have pain? How is the pain the majority of the time? Does this pain limit your usual activities such as household chores or your job?	1=Limiting severe pain	1,003 (7)	0.21 (0.3)
		0.85=Limiting moderate pain	1,088 (7.6)	
		0.68=Limiting mild pain	515 (3.6)	
		0.51=Not limiting severe pain	318 (2.2)	
		0.34=Not limiting moderate pain	1,237 (8.6)	

		0.17=Not limiting mild pain	1,310 (9.1)	
		0=No pain	8,848 (61.7)	
Depressed	During the last week the majority of time felt depressed?	1=Yes	4,977 (34.7)	0.34 (0.47)
		0=No	9,331 (65.2)	
Effort	During the last week the majority of time felt everything you did was difficult to do?	1=Yes	5,087 (35.5)	0.35 (0.47)
		0=No	9,220 (64.4)	
Restless sleep	During the last week the majority of time had restless sleep?	1=Yes	6,005 (41.9)	0.41 (0.49)
		0=No	8,310 (58.1)	
Happy	During the last week the majority of time felt happy?	1=Yes	2,906 (20.3)	0.2 (0.4)
		0=No	11,390 (79.6)	
Enjoyed life	During the last week the majority of time enjoyed life?	1=Yes	3,275 (22.9)	0.2 (0.42)
		0=No	11,019 (77.1)	
Sad	During the last week the majority of time felt sad?	1=Yes	5,797 (40.5)	0.4 (0.49)
		0=No	8,517 (59.5)	
Tired	During the last week the majority of time felt tired?	1=Yes	8,478 (59.2)	0.59 (0.49)
		0=No	5,843 (40.8)	
Energy	During the last week the majority of time felt energetic?	1=Yes	6,876 (48.1)	0.48 (0.49)
		0=No	7,424 (51.9)	
Lost weight	Compared with two years ago, your weight has decreased 5 kilos or more?	1=Yes	4,091 (28.8)	0.28 (0.45)
		0=No	10,126 (71.2)	
Swelling	During the last two years have you frequently had swelling in the feet or ankles?	1=Yes	3,670 (25.6)	0.25 (0.43)
		0=No	10,653 (74.4)	
Respiratory symptoms	During the last two years have you frequently had breathing, panting, coughing or phlegm?	1=Yes	2,571 (17.9)	0.17 (0.38)
		0=No	11,753 (82.1)	

Nausea	During the last two years have you frequently had nausea or fainting?	1=Yes	3,074 (21.4)	0.21 (0.41)
		0=No	11,249 (78.6)	
Fatigue	During the last two years have you frequently had severe fatigue or exhaustion?	1=Yes	3,145 (21.9)	0.21 (0.41)
		0=No	11,176 (78.1)	
Anorexia	In the last two years, have you eaten less because of loss of appetite, digestive problems, and difficulties chewing or swallowing?	1=Often	731 (5.1)	0.17 (0.28)
		0.5=Sometimes	3,484 (24.3)	
		0=Rarely	10,099 (70.6)	
Stomach pain	In the last two years, have you frequently had stomach pain, indigestion or diarrhea?	1=Yes	3,099 (21.6)	0.21 (0.41)
		0=No	11,223 (78.4)	
Urinary incontinence	In the last two years, have you frequently had: incontinence when coughing, sneezing, picking something up, exercising, or when had urge to urinate?	1=Yes	1,268 (8.8)	0.08 (0.28)
		0=No	13,056 (91.2)	
Self-rated grip strength	How would you evaluate your hand strength (your dominant hand)?	1=Very weak	515 (3.6)	0.35 (0.24)
		0.6=Somewhat weak	2,974 (20.7)	
		0.3=Somewhat strong	8.069 (56.3)	
		0=Very strong	2,764 (19.3)	
Self-rated balance	How often do you have difficulty with balance?	1=Often	907 (6.3)	0.24 (0.32)
		0.6=Sometimes	2,625 (18.3)	
		0.3=Rarely	2,481 (17.3)	
		0=Never	8,299 (57.9)	
Rested	How often do you feel really rested when you wake up in the morning?	1=Rarely or never	1,545 (10.7)	0.24 (0.34)
		0.5=Sometimes	4,010 (28)	
		0=Most of the time	8,767 (61.2)	
Self-rated memory	How would you evaluate your memory nowadays?	1=Poor	1,124 (8)	0.63 (0.2)
		0.75=Fai	6,913 (49.2)	

		0.5=Good	4,840 (34.4)	
		0.25=Very good	702 (5)	
		0=Excellent	459 (3.2)	
Self-rated memory compared to two years ago	Compared to the last two years, would you say your memory is?	1=Worse	3,046 (21.7)	0.57 (0.25)
		0.5=More or less the same	10,093 (72.1)	
		0=Better	851 (6)	
Verbal fluency	Names of animals in one minute	Transformation of the score of the verbal fluency test, the highest score (37) was inverted and then a reciprocal was calculated in order to have a 0-1 score		0.77 (0.07)
Orientation	Three orientation questions	1=Three mistakes	680 (4.8)	0.17 (0.27)
		0.6=Two mistakes	922 (6.5)	
		0.3=One mistake	3,355 (24)	
		0=No mistakes	9,024 (64.5)	
Delayed recalling	Test of delayed recalling 8 words	Transformation of the score of the verbal fluency test, the highest score (37) was inverted and then a reciprocal was calculated in order to have a 0-1 score		
Walking blocks	Because of a health problem, do you have difficulty walking several blocks?	1=Can´t do/doesn´t do	105 (0.7)	0.14 (0.23)
		0.5=Yes	3,793 (26.7)	
		0=No	10,317 (72.6)	
Running/jogging	Because of a health problem, do you have difficulty running or jogging one kilometer?	1=Can´t do/doesn´t do	5,842 (41.3)	0.51 (0.44)
		0.5=Yes	2,808 (19.8)	
		0=No	5,516 (38.9)	
Sitting	Because of a health problem, do you have difficulty sitting for about two hours?	1=Can´t do/doesn´t do	67 (0.5)	0.1 (0.2)
		0.5=Yes	2,733 (19.2)	
		0=No	11,416 (80.3)	
Getting up		1=Can´t do/doesn´t do	36 (0.3)	0.15 (0.23)

	Because of a health problem, do you have difficulty getting up from a chair after sitting for long periods?	0.5=Yes	4,253 (29.9)	
		0=No	9,928 (69.8)	
Climbing stairs	Because of a health problem, do you have difficulty climbing several flights of stairs without resting?	1=Can´t do/doesn´t do	1,242 (8.8)	0.28 (0.32)
		0.5=Yes	5,593 (39.4)	
		0=No	7,352 (51.8	
Stooping/kneeling/crouching	Because of a health problem, do you have difficulty stooping, kneeling, or crouching?	1=Can´t do/doesn´t do	210 (1.5)	0.21 (0.26)
		0.5=Yes	5,713 (40.2)	
		0=No	8,293 (58.3)	
Extending arms	Because of a health problem, do you have difficulty reaching or extending your arms above shoulder level?	1=Can´t do/doesn´t do	36 (0.3)	0.06 (0.17)
		0.5=Yes	1,877 (13.2)	
		0=No	12,304 (86.5)	
Pulling/pushing	Because of a health problem, do you have difficulty pulling or pushing large objects like a living-room chair?	1=Can´t do/doesn´t do	395 (2.8)	0.14 (0.25)
		0.5=Yes	3,313 (23.3)	
		0=No	10,508 (73.9)	
Lifting objects	Because of a health problem, do you have difficulty lifting or carrying objects that weigh over 5kg, like a heavy bag of groceries?	1=Can´t do/doesn´t do	307 (2.2)	0.13 (0.24)
		0.5=Yes	3,234 (22.7)	
		0=No	10,672 (75.1)	
Picking	Because of a health problem, do you have difficulty picking up a coin from the table?	1=Can´t do/doesn´t do	39 (0.3)	0.03 (0.13)
		0.5=Yes	982 (6.9)	
		0=No	13,195 (92.8)	
Dressing *	Because of a health problem, do you have difficulty dressing including putting on shoes and socks?	1=Can´t do/doesn´t do	30 (0.2)	0.05 (0.15)
		0.5=Yes	1,279 (9)	
		0=No	12,905 (90.8)	
Walking in a room*		1=Can´t do/doesn´t do	22 (0.2)	0.03 (0.12)

	Because of a health problem, do you have difficulty walking across a room?	0.5=Yes	744 (5.2)	
		0=No	13,451 (94.6)	
Bathing *	Because of a health problem, do you have difficulty bathing or showering?	1=Can´t do/doesn´t do	31 (0.2)	0.02 (0.09)
		0.5=Yes	415 (2.9)	
		0=No	13,741 (96.9)	
Eating *	Because of a health problem, do you have difficulty eating, such as cutting your food?	1=Can´t do/doesn´t do	51 (0.4)	0.01 (0.09)
		0.5=Yes	280 (1.9)	
		0=No	13,856 (97.7)	
Going to bed *	Because of a health problem, do you have difficulty getting into or out of bed?	1=Can´t do/doesn´t do	13 (0.1)	0.15 (0.23)
		0.5=Yes	960 (6.8)	
		0=No	13,214 (93.1)	
Using the toilet *	Because of a health problem, do you have difficulty using the toilet, including getting on and off the toilet or squatting?	1=Can´t do/doesn´t do	28 (0.2)	0.02 (0.11)
		0.5=Yes	651 (4.6)	
		0=No	13,508 (95.2)	
Preparing a meal	Because of a health problem, do you have difficulty preparing a hot meal?	1=Can´t do/doesn´t do	712 (5)	0.07 (0.23)
		0.5=Yes	509 (3.6)	
		0=No	12,995 (91.4)	
Shopping	Because of a health problem, do you have difficulty shopping for groceries?	1=Can´t do/doesn´t do	388 (2.7)	0.06 (0.2)
		0.5=Yes	1,090 (7.7)	
		0=No	12,736 (89.6)	
Taking medicines	Because of a health problem, do you have difficulty taking medications (if you take any or needed to do so)?	1=Can´t do/doesn´t do	171 (1.2)	0.02 (0.12)
		0.5=Yes	305 (2.2)	
		0=No	13,741 (96.6)	
Managing money		1=Can´t do/doesn´t do	52 (0.4)	0.01 (0.09)

	Because of a health problem, do you have difficulty managing your money?	0.5=Yes	310 (2.2)	
		0=No	13,853 (97.4)	

*Activities of daily living

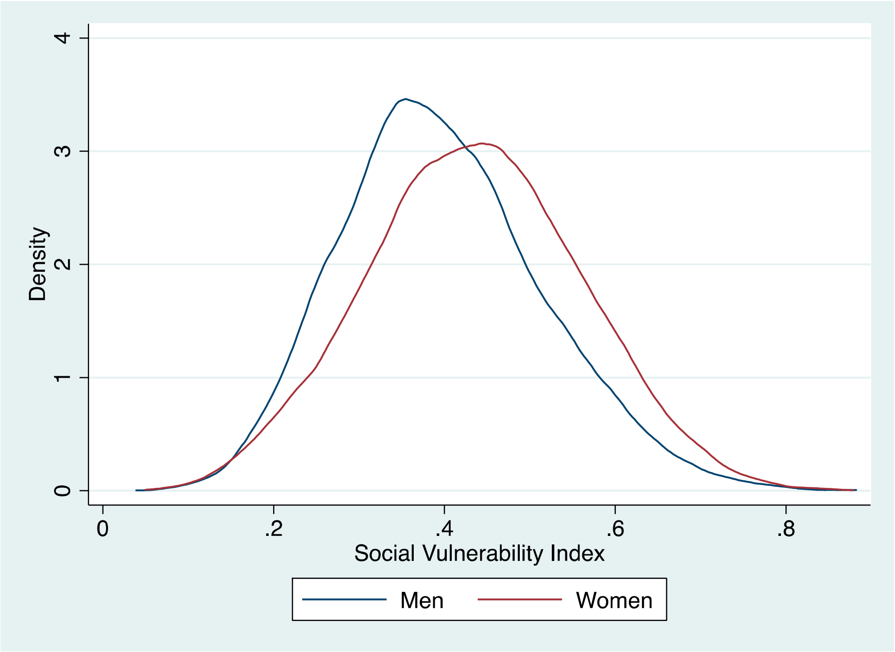

Supplementary figure 21: Kernel density of the social vulnerability index stratified for sex

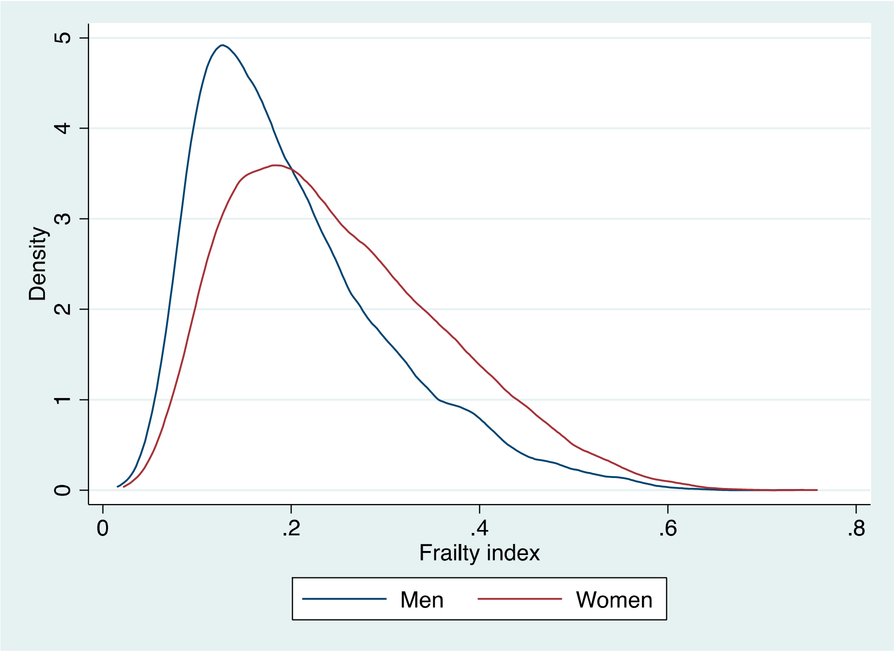

Supplementary figure 2. Kernel distribution of the frailty index according to sex

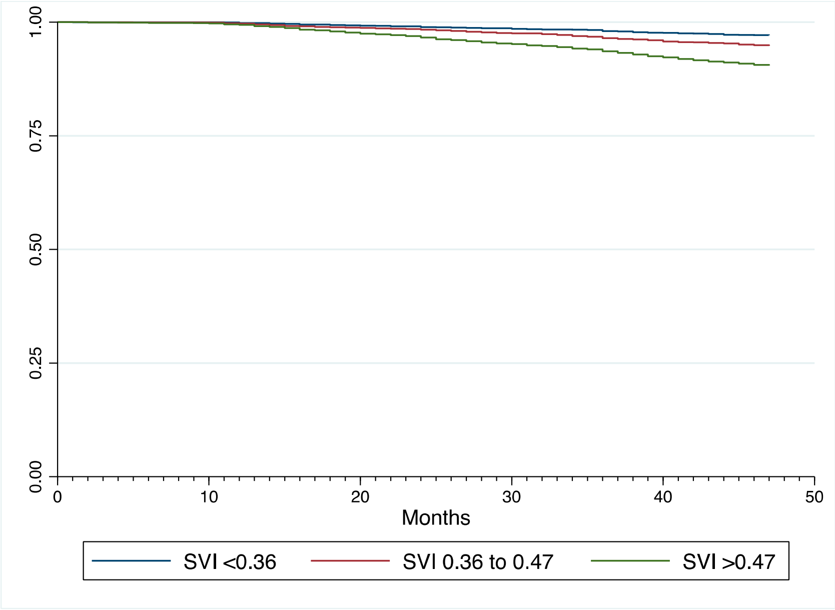

Supplementary Figure 3: Kaplan-Meier curves for social vulnerability index score tertiles

Formatted: Font: (Default) Arial, 12 pt

Formatted: Font: (Default) Arial, 12 pt

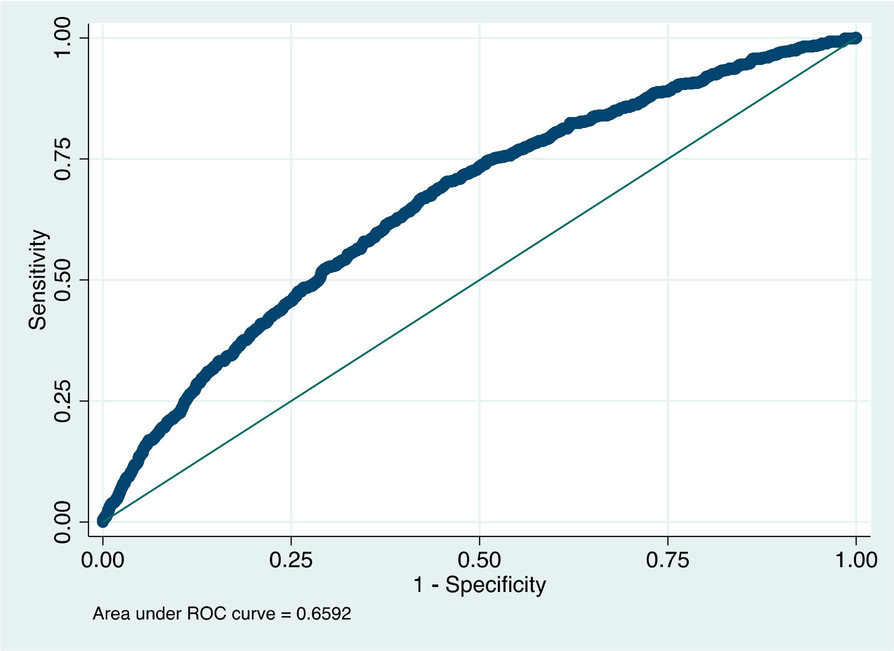

Supplementary Figure 4: ROC curve for the social vulnerability index on mortality

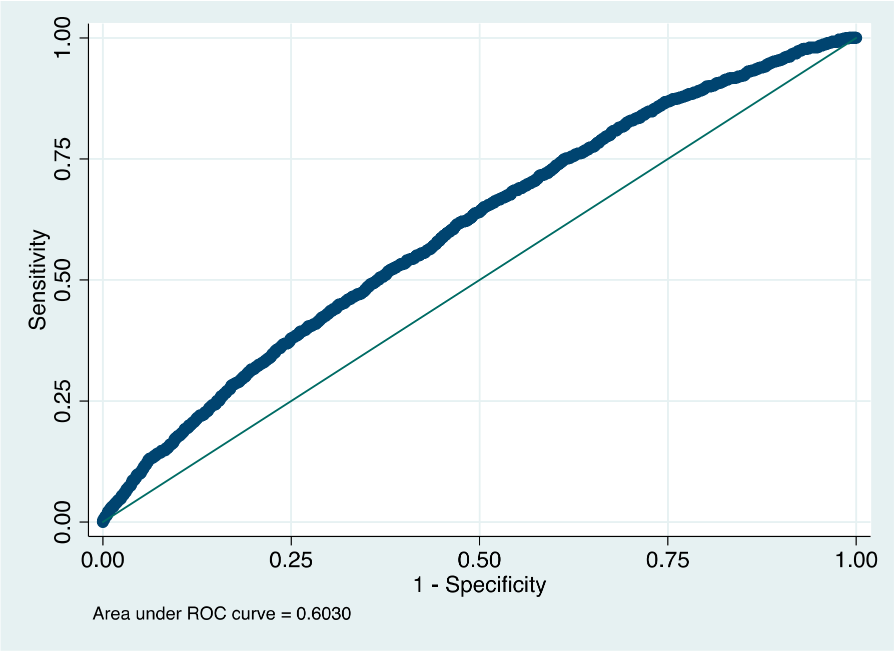

Supplementary Figure 54: ROC curve for the social vulnerability index on incident disability

Supplementary Table 3: Cox and logistic regressions (mortality and incident disability respectively) fully adjusted with interaction terms between frailty index and social vulnerability index

	Mortality			Incident disability		
	HR	95% CI	p-value	OR	95% CI	p-value
SVI	8.4	1.9-36.9	0.005	3.5	1-13.6	0.05
FI	364.8	39.6-3353	<0.001	5234	516.2-5306.1	<0.001
Sex	0.5	0.4-0.6	<0.001	0.89	0.77-1.03	0.125
Age	1.1	1.1-1.2	<0.001	1.04	1.3-1.05	<0.001
Physically active	0.6	0.5-0.8	<0.001	0.8	0.7-0.9	0.001
Tobacco use: Never-reference						
Former user	1	0.8-1.2	0.89	0.9	0.8-1.1	0.332
Current user	1.1	0.8-1.4	0.38	0.9	0.7-1.1	0.319
High-risk alcohol drinking	0.7	0.5-1.1	0.183	1.1	0.9-1.4	0.161
FI*SVI	0.01	0.01-1.4	0.072	0.01	0.01-2.6	0.116

Supplementary Table 4. Bivariate analysis of mortality. Women

Formatted: English (United States)

	Alive (n=7,390)	Dead (n=388)	p-value *
Age categories, n (%)			
40-49	522 (7.1)	3 (0.6)	<0.001
50-59	2,518 (34.1)	33 (8.5)	
60-69	2,546 (34.5)	106 (27.3)	
70-79	1,378 (18.7)	122 (31.4)	
≥80	426 (5.8)	124 (32)	
Physically active, n (%)	2,496 (33.8)	66 (17)	<0.001
Tobacco use, n (%)			
Never	5,874 (79.5)	330 (85.1)	0.001
Former user	981 (13.3)	48 (12.3)	
Current user	535 (7.2)	10 (2.6)	
High-risk alcohol drinking, n (%)	283 (3.8)	4 (1)	0.004
Social vulnerability index, mean (SD)	0.43 (0.12)	0.51 (0.1)	<0.001
Frailty index, mean (SD)	0.24 (0.1)	0.34 (0.13)	<0.001

Supplementary Table 5. Bivariate analysis of mortality. Men

	Alive (n=5,118)	Dead (n=397)	p-value *
Age categories, n (%)			
40-49	99 (1.9)	1 (0.2)	<0.001
50-59	1,469 (28.7)	24 (6.1)	
60-69	2,070 (40.4)	120 (30.2)	
70-79	1,110 (21.7)	140 (35.2)	
≥80	370 (7.2)	112 (28.2)	
Physically active, n (%)	2,531 (49.4)	111 (27.9)	<0.001
Tobacco use, n (%)			
Never	2,037 (39.8)	144 (36.3)	0.159
Former user	2,095 (40.9)	182 (45.8)	
Current user	986 (19.3)	71 (17.9)	
High-risk alcohol drinking, n (%)	910 (17.8)	35 (8.8)	<0.001
Social vulnerability index, mean (SD)	0.39 (0.1)	0.46 (0.12)	<0.001
Frailty index, mean (SD)	0.19 (0.09)	0.28 (0.12)	<0.001

Supplementary Figure 6. Kaplan Meier for women,

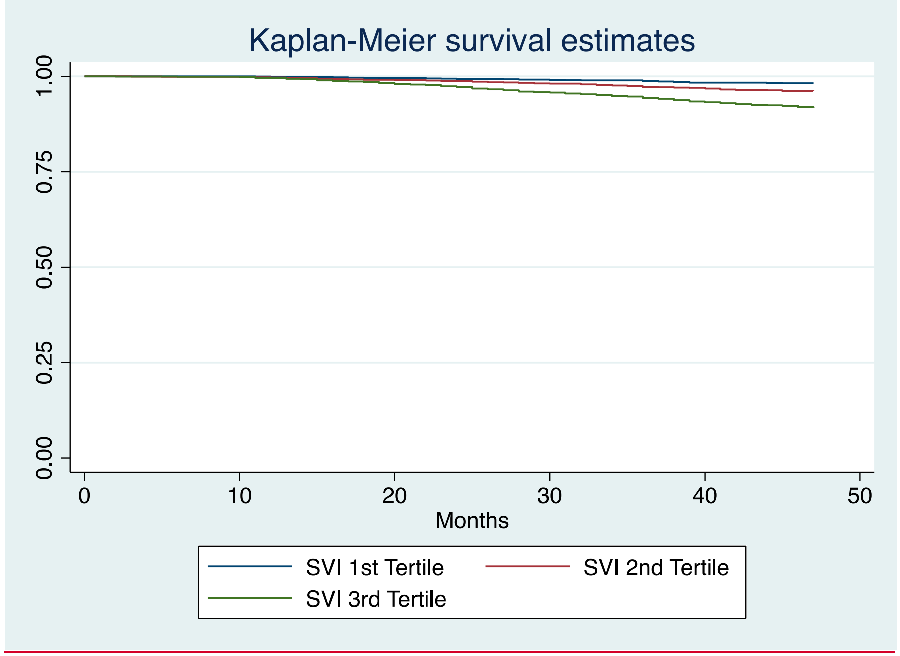

Supplementary Figure 7. Kaplan Meier for men,

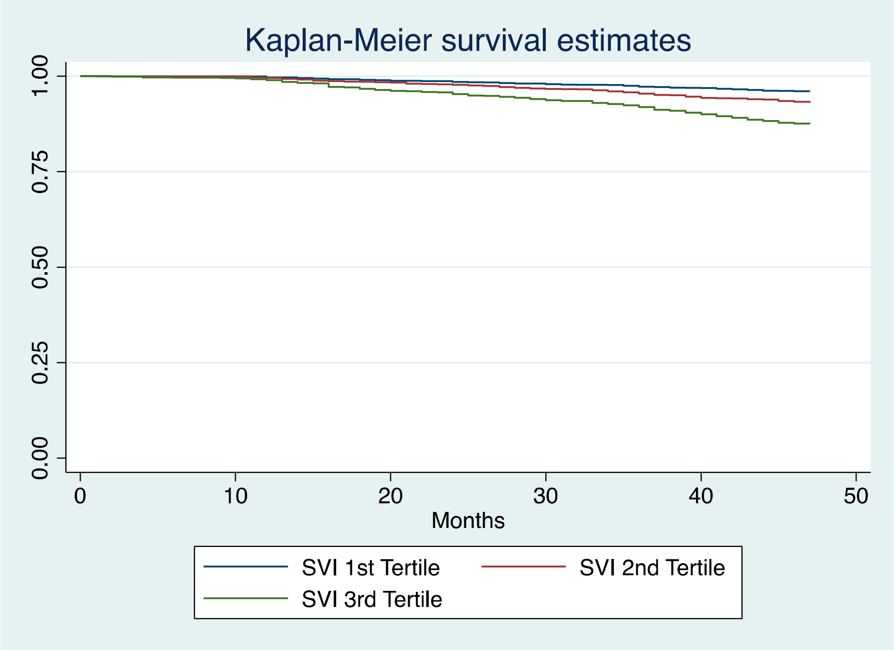

Supplementary Table 6. Bivariate analysis of disability. Women

	No disability (n=5,069)	Disability (n=828)	p-value *
**Age categories, n (%)**			
**40-49**	432 (8.5)	29 (3.5)	<0.001
**50-59**	2,001 (39.5)	196 (23.7)	
**60-69**	1,714 (33.8)	308 (37.2)	
**70-79**	760 (14.9)	217 (26.2)	
**≥80**	162 (3.2)	78 (9.4)	
**Physically active, n (%)**	1,894 (37.4)	227 (27.4)	<0.001
**Tobacco use, n (%)**			
**Never**	4,024 (79.4)	675 (81.5)	0.363
**Former user**	662 (13.1)	96 (11.6)	
**Current user**	383 (7.6)	57 (6.9)	
**High-risk alcohol drinking, n (%)**	214 (4.2)	27 (3.3)	0.195
**Social vulnerability index, mean (SD)**	0.41 (0.12)	0.46 (0.12)	<0.001
**Frailty index, mean (SD)**	0.21 (0.09)	0.27 (0.1)	<0.001

Supplementary Table 7. Bivariate analysis of disability. Men

	No disability (n=3,881)	Disability (n=539)	p-value *
**Age categories, n (%)**			
**40-49**	85 (2.2)	5 (0.9)	<0.001
**50-59**	1,309 (33.7)	94 (17.4)	
**60-69**	1,592 (41)	213 (39.5)	
**70-79**	725 (18.7)	154 (28.6)	
**≥80**	170 (4.4)	73 (13.5)	
**Physically active, n (%)**	2,030 (52.3)	228 (42.3)	<0.001
**Tobacco use, n (%)**			
**Never**	1,579 (40.7)	227 (42.1)	0.059
**Former user**	1,516 (39.1)	226 (41.9)	
**Current user**	786 (20.3)	86 (15.9)	
**High-risk alcohol drinking, n (%)**	748 (19.3)	89 (16.5)	0.125
**Social vulnerability index, mean (SD)**	0.38 (0.1)	0.42 (0.1)	<0.001
**Frailty index, mean (SD)**	0.16 (0.07)	0.23 (0.09)	<0.001

Supplementary Table 8. Multivariate analysis of mortality and incident disability. Women

	Mortality						Incident disability					
	Model 1			Model 2			Model 1			Model 2		
	HR	95% CI	p-value	HR	95% CI	p-value	OR	95% CI	p-value	OR	95% CI	p-value
**Social vulnerability index tertiles**												
**<0.36**	Reference											
**0.36-0.47**	2.2	1.5-3.2	<0.001	1.2	0.9-1.5	0.054	1.5	1.2-1.8	<0.001	1.07	0.87-1.3	0.487
**>0.47**	4.8	3.4-6.7	<0.001	1.4	1.1-1.8	<0.001	2.4	1.9-2.9	<0.001	1.2	0.97-1.4	0.078
**Age categories**												
**40-49**	Reference											
**50-59**				1.9	0.7-5.3	0.21				1.26	0.84-1.9	0.258
**60-69**				5.4	2-14.5	0.001				2.13	1.42-3.2	<0.001
**70-79**				9.2	3.4-24.9	<0.001				2.97	1.9-4.52	<0.001
**≥80**				19.1	7.1-51.7	<0.001				4.34	2.7-7.05	<0.001
**Physically active**				0.6	0.5-0.7	<0.001				0.78	0.6-0.92	0.005
**Tobacco use**												
**Never**	Reference											
**Former user**				0.99	0.83-1.18	0.84				0.87	0.68-1.1	0.256
**Current user**				1.04	0.81-1.3	0.81				1.01	0.74-1.3	0.93
**High-risk alcohol drinking**				0.7	0.5-1	0.11				1.12	0.73-1.7	0.596

**Frailty index levels**												
**<0.1**	Reference											
**0.11-0.2**				1.2	0.7-1.9	0.05				2.19	1.23-3.9	0.007
**0.21-0.3**				1.5	0.9-2.3	0.092				4.39	2.4-7.8	<0.001
**>0.31**				2.7	1.7-4.2	<0.001				8.6	4.8-15.2	<0.001

Supplementary Table 9. Multivariate analysis of mortality and incident disability. Men

	**Mortality**						**Incident disability**					
	Model 1			Model 2			Model 1			Model 2		
	HR	95% CI	p-value	HR	95% CI	p-value	OR	95% CI	p-value	OR	95% CI	p-value
**Social vulnerability index tertiles**												
**<0.36**	Reference											
**0.36-0.47**	1.8	1.3-2.3	<0.001	1.18	0.9-1.56	0.218	1.7	1.4-2.1	<0.001	1.23	0.98-1.5	0.068
**>0.47**	3.3	2.5-4.2	<0.001	1.48	1.12-1.95	0.006	1.9	1.5-2.4	<0.001	1.06	0.81-1.3	0.662
**Age categories**												
**40-49**	Reference											
**50-59**				1.58	0.21-11.7	0.65				1.15	0.45-2.9	0.768
**60-69**				4.63	0.64-33.2	0.127				1.85	0.73-4.6	0.191
**70-79**				7.71	1.07-55.4	0.042				2.4	0.96-6.2	0.06

**≥80**		13.5	1.87-97.5	0.01	4.2	1.6-10.9	0.004
**Physically active**		0.61	0.48-0.76	<0.001	0.8	0.67-0.9	0.029
**Tobacco use**							
**Never**	Reference						
**Former user**		1.09	0.8-1.36	0.415	0.92	0.75-1.1	0.492
**Current user**		1.28	0.96-1.71	0.086	0.81	0.61-1.1	0.143
**High-risk alcohol drinking**		0.78	0.54-1.1	0.176	1.13	0.8-1.46	0.342
**Frailty index levels**							
**<0.1**	Reference						
**0.11-0.2**		1.23	0.74-2.07	0.412	1.63	1.09-2.4	0.017
**0.21-0.3**		1.54	0.91-2.61	0.108	3.72	2.45-5.6	<0.001
**>0.31**		3.15	1.87-5.31	<0.001	7.16	4.6-11.2	<0.001
